# Ultrasonic plasticizing micro-injection molding of UHMWPE based on new process flow and ultrasonic system structure to improve mechanical properties and process stability

**DOI:** 10.1016/j.ultsonch.2025.107272

**Published:** 2025-02-12

**Authors:** Zhiying Shan, Xingbo Qin, Hang Li, Yanghui Xiang, Wangqing Wu

**Affiliations:** a State Key Laboratory of Precision Manufacturing for Extreme Service Performance, Central South University, Changsha 410083, China; b School of Mechanical and Electrical Engineering, Central South University, Changsha 410083, China; c School of Mechanical and Electrical Engineering, Changsha University, Changsha 410022, China

**Keywords:** UHMWPE, Ultrasonic plasticizing micro-injection molding, Ultrasonic system stability, Mechanical property, Ultrasonic degradation

## Abstract

Ultrasonic plasticizing micro-injection molding (UPMIM) technology has been considered as an effective means of UHMWPE molding. However, the cumbersome forming process, the degradation of mechanical properties and the poor consistency of molding and property seriously restrict further application. In this study, a new ultrasonic molding method of UHMWPE micro-parts is proposed. Firstly, the UHMWPE ultrasonic plasticizing material was prepared simply and quickly by ultrasonic technology. Secondly, the UHMWPE tensile samples were molded by an innovative UPMIM structure with a large diameter ratio of the ultrasonic sonotrode to plasticizing cavity. Then, the optimum molding process parameters were obtained by grey relational analysis (GRA). After that, the influence of system stability and process parameters on mechanical properties and consistency was studied by contribution analysis. Finally, compared with the typical UHMWPE molding method (compression molding) and the existing research results, the influence and feasibility of the process are analyzed in detail. The results show that the ultrasonic technique can effectively prepare UHMWPE tablets with almost unchanged properties (molecular weight decreased by 0.31 %). A large diameter ratio of the ultrasonic sonotrode to plasticizing cavity can expand the process window for complete filling of UHMWPE tensile samples, and the filling stability of the ultrasonic system is increased by about 1.8 times. Meanwhile, this ultrasonic system structure can also inhibit the oxidative degradation of UHMWPE, reduce the break of molecular chain. The elongation at break (EB) of tensile samples increased from 5.56 % to 12.2 %, while the tensile strength (TS) decreases from 136.54 % to 68.11 %. Moreover, the contribution of process parameters to the mechanical properties and consistency for UHMWPE tensile samples is 55.97 %–88.37 %, while the contribution of ultrasonic system stability is 11.63 %–44.03 %.

## 1 Introduction

Ultrahigh molecular weight polyethylene (UHMWPE) is a linear polyethylene with a molecular weight of more than 1.5 million branchless chains, which is a polymer thermoplastic material with excellent properties. As a thermoplastic material, UHMWPE boasts an array of exceptional properties, including robust impact resistance, superb self-lubrication, stable chemical attributes, and high resistance to aging. These features make it invaluable across diverse sectors such as industrial engineering, national defense, and healthcare [1], [2]. With the rapid advancements in micro-system technology (MST) and micro-electro-mechanical systems (MEMS), the demand for UHMWPE micro-components is experiencing significant growth [3]. Current micro-forming technologies encompass methods like injection molding [4], injection compression molding [5], and hot-embossing [6].

However, the high viscosity, poor fluidity, and low critical shear rate of UHMWPE melt render conventional polymer micro-forming processes unsuitable. Initially, researchers employed injection molding and injection compression molding techniques to molding UHMWPE micro-parts. It was observed that injection compression molding offered superior reproduction and molding precision for micro-parts. Nevertheless, this process demands significant specifications from the injection molding machine (clamping force 50 T, maximum injection speed 330 mm/s) and raw material (injection grade UHMWPE) [7], [8]. Subsequently, ultrasonic coining technology was introduced for molding UHMWPE micro-parts, such as micro-gears [9], micro-column arrays [10], and hydrophobic surface micro-parts [11]. In this process, UHMWPE powder is initially placed into the mold cavity, where it is compacted and plasticized by the ultrasonic sonotrode. The friction and viscoelastic heat generated facilitate the fusion of the powder particles, forming various micro-parts. Although this method achieves high molding precision, it is prone to defects like cracks and trapped gas. Additionally, the inherent characteristics of ultrasonic coining significantly restrict its application scenarios. Therefore, there is a pressing need to develop a more versatile micro-forming technology to adequately address the molding requirements of UHMWPE micro-parts.

Ultrasonic plasticizing micro-injection molding (UPMIM) technology is a new type of micro-injection molding technology proposed by researchers based on the successful experience of power ultrasound in polymer molding [12], [13], [14]. This technique harnesses ultrasonic vibrations to melt the polymer. Then, the molten polymer is propelled into the mold cavity by a plunger, culminating in the production of micro-scaled, high-precision components. As a kind of microinjection molding, UPMIM not only inherits the advantages of low cost, batch production and high molding accuracy of microinjection molding technology, but also has the characteristics of low-pressure molding and low energy consumption. Meanwhile, the unique direct pressure injection structure can effectively solve the problem of material waste in micro-injection molding [15], [16]. Empirical studies validate that UPMIM effectively fills micro-scaled cavities and easily molds various high aspect ratio micro-structures, such as microneedle arrays [17] and prism arrays [18]. Moreover, it demonstrates strong adaptability, successfully molding materials across disparate shapes-like particles [19] and rods [20]-and diverse polymer types including polypropylene (PP) [21], polylactide (PLA) [22], cyclic olefin copolymer (COC) [23], polyphenylsulfone (PPSU) [24], and polyetheretherketone (PEEK) [25].

To this end, researchers have explored the application of UPMIM technology to the fabrication of UHMWPE micro-parts, successfully molding micro-tensile samples. It was observed that UHMWPE powder is unsuitable for direct use in UPMIM, necessitating the use of compression molding molds and professional compression molding equipment to make ultrasonic plasticizing materials. Meanwhile, the ultrasonic molding of UHMWPE micro-tensile samples demands large amplitude and high mold temperature (exceeding 100℃). Furthermore, the process is characterized by a strong coupling between ultrasonic plasticization and plunger injection, resulting in instability during the molding of UHMWPE micro-tensile samples [26]. In experiments involving UHMWPE micro-tensile samples with varying graphite content, the addition of 1 wt% graphite enhanced tensile strength by 8.8 %, yet the elongation at break was a mere 0.2 %. Excessive ultrasonic action can lead to rapid oxidative degradation and chain scission of UHMWPE [27]. In summary, there are also several challenges in the ultrasonic molding of UHMWPE: (1) the process is complex and cumbersome; (2) the product mechanical property is seriously decreased; and (3) the consistency of the molding process is poor.

To address the complexity of the molding process, ultrasonic technology was applied to melt UHMWPE powder by generating an ultrasonic energy field and pressure field, creating UHMWPE tablets that can be directly molded through ultrasonic plasticization. To mitigate issues of product for performance degradation and poor consistency, the structure of the ultrasonic system was optimized. Specifically, a large diameter ratio of the ultrasonic sonotrode to plasticizing cavity was employed to increase the contact area between the sonotrode and the molten material. This approach not only enhances plasticization and filling efficiency but also reduces the residence time of the melt within the ultrasonic energy field, thereby lowering the risk of oxidative degradation. Meanwhile, the larger diameter of the ultrasonic sonotrode improves system rigidity, minimizes energy field fluctuations during molding, and enhances both the consistency of the molding process and the stability of the material properties.

Furthermore, the molding of UHMWPE needs to consider multiple evaluation indexes, which is a typical multi-objective optimization problem. In the realm of multi-objective optimization, grey relational analysis (GRA) is widely employed across various domains due to its simplicity and strong applicability [28], [29], [30]. This method, introduced by Professor Deng in 1982 [31], is a multi-factor statistical analysis method designed to deal with problems characterized by incomplete, inaccurate, and uncertain information. It can effectively evaluate multiple response indicators and quickly screen out the optimal scheme [32], [33].

The significance of this study is multifaceted: (1) It introduces a novel process flow for ultrasonic molding of UHMWPE, aimed at simplifying the molding complexity; (2) It incorporates an enhanced ultrasonic process structure featuring a large diameter ratio of the ultrasonic sonotrode to the plasticizing cavity, resulting in micro-parts with improved performance and consistency; (3) It optimizes process parameters and quantitatively assesses the impact of process system stability on performance consistency using GRA and contribution analysis. This research provides a foundational basis and a point of reference for future developments in ultrasonic molding of UHMWPE.

This study is composed of several distinct phases: initially, UHMWPE ultrasonic plasticizing material was prepared using the newly proposed process. Subsequently, an experiment focused on the ultrasonic process was undertaken to mold tensile samples of UHMWPE, utilizing a large diameter ratio of the ultrasonic sonotrode to the plasticizing cavity. During this phase, a multi-objective optimization of the process parameters was also conducted. After that, contribution analysis was performed to assess the effects of system stability and process parameters on the molding quality, material properties, and consistency. Lastly, the performance of the tensile samples was evaluated adopting a variety of characterization techniques, confirming the feasibility of the proposed method.

## 2 Research method of ultrasonic molding for UHMWPE powder

**Fig. 1:**
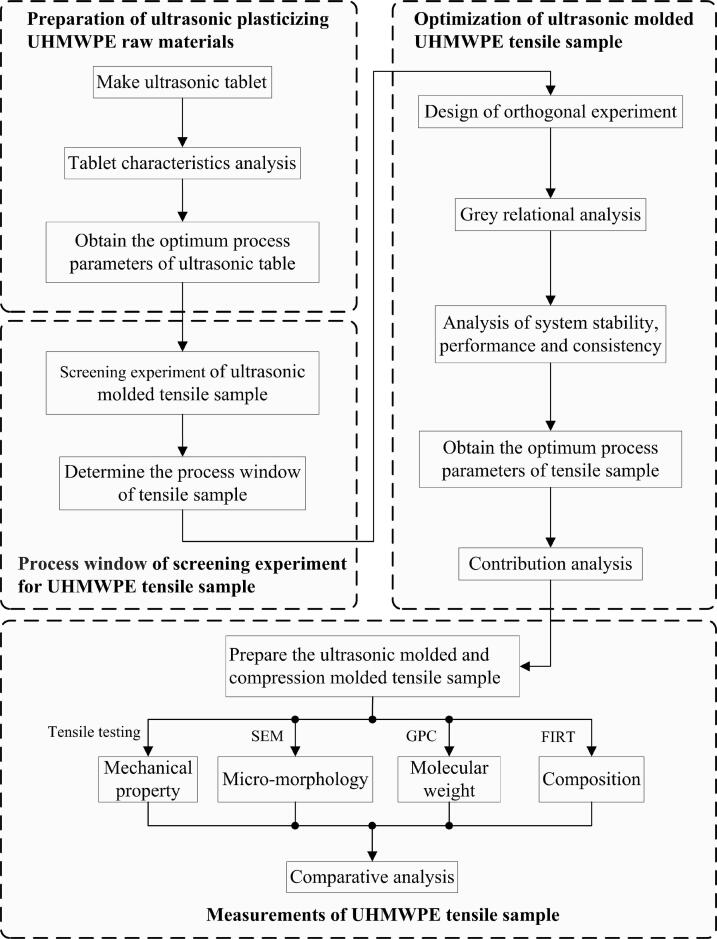
Flowchart of research method for ultrasonic molded UHMWPE tensile sample.

This study encompasses three integral components: the preparation of ultrasonic plasticized UHMWPE raw materials, the optimization of ultrasonic plasticized UHMWPE tensile samples, and the measurements of properties for UHMWPE tensile samples, as shown in Fig. 1.

Firstly, the preparation of ultrasonic plasticizing raw materials: ① The UHMWPE tablet was fabricated by ultrasonic technology. ② The influence of different process parameters on the weight of the tablet was explored, and the process parameters corresponding to the maximum weight of the tablet were selected as the best process for ultrasonic tablet manufacturing. ③ The ultrasonic table was processed into irregular shaped particles to complete the preparation of ultrasonic plasticizing raw materials. Secondly, optimization of ultrasonic plasticized UHMWPE tensile sample: ① According to the process window of UHMPE tensile sample preforming experiment, the range of ultrasonic process parameters was determined. ② The orthogonal experiment method was applied to design the experiment. ③ Multi-objective optimization was carried out by GRA method to obtain the optimum process parameters of the tensile sample. ④ The influence of process parameters on the ultrasonic system stability, mechanical property and consistency was analyzed. Thirdly, measurements of UHMWPE tensile samples: The mechanical properties, microstructure, molecular weight and composition of UHMWPE tensile samples molded under optimal ultrasonic parameters and compression molded tensile samples were measured. Then, the differences and feasibility of these two UHMWPE forming techniques were compared and analyzed in detail.

## 3 Experiment, methods and measurements

### 3.1 Experiment

#### 3.1.1 Material

The experimental material is UHMWPE powder with grade 1050 produced by Celanese company. The average molecular weight is 5*10^6^ g/mol and the powder diameter is 100 μm. The powder density was 0.936 g/cm^3^ (23 ℃), and the melting index was less than 0.1 g/min (230 °C, 21.6 N).

#### 3.1.2 Ultrasonic molding equipment and tensile sample geometry

**Fig. 2:**
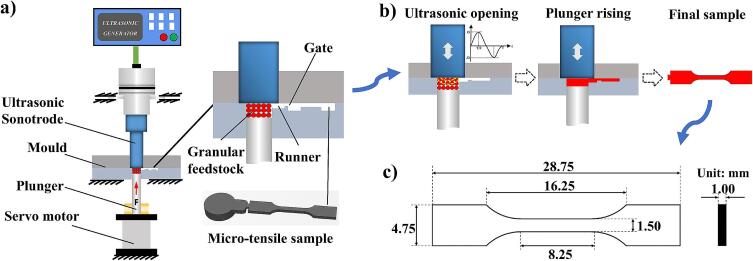
The UPMIM equipment, principle and part geometry. (a) The UPMIM equipment structure. (b) The principle and process for UPMIM. (c) The geometry of the tensile sample.

The self-developed UPMIM equipment was adopted to shape UHMWPE products. The performance parameters of the equipment are shown in Table 1. Among them, the ultrasonic frequency is 30 kHz, the maximum clamping force is 1.2 T, the maximum plunger speed is 12 mm/s, the diameter of the ultrasonic sonotrode is 15 mm, and the diameter of the plasticizing cavity is 10 mm (the diameter ratio of the ultrasonic sonotrode to the plasticizing cavity is 15 mm:10 mm). The equipment mainly includes: ultrasonic generator, ultrasonic sonotrode, mould, plunger, plunger servo motor, as shown in Fig. 2a.

**Table 1:** Performance parameters of UPMIM equipment.

Project	Parameter
Ultrasonic frequency	30 k Hz
Maximum clamping force	1.2T
Maximum plunger speed	12 mm/s
Ultrasonic sonotrode diameter	15 mm
Plasticizing cavity diameter	10 mm

According to ASTM D638 plastic tensile test type IV components, the UHMWPE tensile samples were prepared, and the feasibility of the process was evaluated by characterizing the performance of the samples. In addition, due to the micro-forming process, the size of the standard tensile sample was reduced by an equal proportion of 4:1, and the geometry is shown in Fig. 2c.

### 3.2 Methods

#### 3.2.1 The raw material preparation and molding methods of UHMWPE

**Fig. 3:**
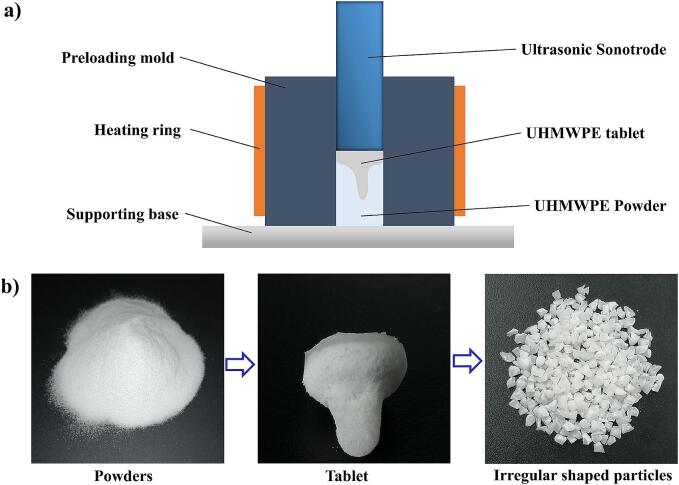
The ultrasonic molding principle of UHMWPE tablet and preparation process of UHMWPE ultrasonic plasticizing raw material. (a) The device and principle of making ultrasonic tablet. (b) The whole process of UHMWPE from powder to tablet and then to irregular particle.

In this study, a new method for preparing UHMWPE tablet using UPMIM equipment was proposed, which can replace the original complicated compression molding process and prepare ultrasonic plasticizing raw materials quickly and conveniently. The principle of ultrasonic tablet preparation is shown in Fig. 3a. The device mainly includes: ultrasonic sonotrode, tablet mold, heating ring and support seat. The process flow of ultrasonic plasticizing raw material preparation is as follows: ① Heat the tablet mould through the heating ring to make the mould have a certain initial temperature. ② Add a quantitative amount of UHMWPE powder into the plasticizing chamber of the tablet mold. ③ Extend the ultrasonic sonotrode into the plasticizing chamber for a certain distance and apply pressure to the powder. ④ Turn on ultrasound, press and form. ⑤Treat the ultrasonic tablet into irregular shape particles. Fig. 3b shows the whole process of UHMWPE from powder to ultrasonic tablet and then to ultrasonic plasticizing raw material.

Fig. 2b shows the principle and main process of UPMIM: First, the polymer is melted and plasticized by ultrasonic vibration hammering. Then, the plunger is adopted to push the melt into the mold cavity. Finally, the product is ejected from the mold. So as to realize the injection molding of micro-size and high-precision parts.

#### 3.2.2 Grey relational analysis method

In this study, the multi-objective optimization method of GRA was applied to optimize the process of ultrasonic molding UHMWPE tensile sample. The GRA method and process are as follows: [34], [35].

**Step 1**: Data normalization

If the index of the original sequence is “the larger the better”, the following formula is used for normalization:

(1) $$x_{i}^{*}\left(t\right)=\frac{x_{i}\left(t\right)-Maxx_{i}\left(t\right)}{\mathrm{Max}x_{i}\left(t\right)-Minx_{i}\left(t\right)}$$

If the index of the original sequence is “the small the better”, the following formula is used for normalization:

(2) $$x_{i}^{*}\left(t\right)=\frac{\mathrm{Max}x_{i}\left(t\right)-x_{i}\left(t\right)}{\mathrm{Max}x_{i}\left(t\right)-Minx_{i}\left(t\right)}$$

If the index of the original sequence is the target value, the following formula is used for normalization:

(3) $$x_{i}^{*}\left(t\right)=1-\frac{\left|x_{i}\left(t\right)-A\right|}{MAX\left\{Maxx_{i}\left(t\right)-A,A-Minx_{i}\left(t\right)\right\}}$$

where $x_{i}^{*}\left(t\right)$ is the normalized sequence, $x_{i}\left(t\right)$ represents the *i* experimental result in the *t* sequence, $\mathrm{Max}x_{i}\left(t\right)$ denotes the maximum value of the experimental result in the *t* sequence, $\mathrm{Min}x_{i}\left(t\right)$ denotes the minimum value of the experimental result in the *t* sequence, i=1,2,3,…m_1_, t = 1,2,3,…m_2_, m_1_ represents the number of experimental results in the same sequence, m_2_ represents the number of sequences.

**Step 2**: Calculate the grey relation coefficient (GRC).

The grey relation coefficient can be calculated as follows:

(4) $$\gamma \left(x_{r}^{*}\left(t\right),x_{i}^{*}\left(t\right)\right)=\frac{\Delta_{\min}+\omega \Delta_{\max}}{\Delta_{\mathrm{ri}}\left(t\right)+\omega \Delta_{\max}}$$

where $\gamma \left(x_{r}^{*}\left(t\right),x_{i}^{*}\left(t\right)\right)$ denotes the grey relation coefficient, $x_{r}^{*}\left(t\right)$ is the reference value of t sequence, usually takes the value of 1. ω is the difference coefficient, ranging from 0 to 1, usually takes the value of 0.5. $\Delta_{\mathrm{ri}}\left(t\right)$, $\Delta_{\min}$ and $\Delta_{\max}$ can be deduced as below:

(5) $$\Delta_{\mathrm{ri}}\left(t\right)=\left|x_{r}^{*}\left(t\right)-x_{i}^{*}\left(t\right)\right|$$

(6) $$\Delta_{\min}=\underset{\forall i}{\underbrace{\min}}\underset{\forall t}{\underbrace{\min}}\Delta_{\mathrm{ri}}\left(t\right)$$

(7) $$\Delta_{\max}=\underset{\forall i}{\underbrace{\max}}\underset{\forall t}{\underbrace{\max}}\Delta_{\mathrm{ri}}\left(t\right)$$

**Step 3**: Calculate the grey relational grade (GRG)

If the importance of each sequence is the same, the average GRC is used to obtain the GRG:

(8) $$\varphi \left(x_{r}^{*},x_{i}^{*}\right)=\frac{1}{m_{2}}\sum_{t=1}^{m_{2}}\gamma \left(x_{r}^{*}\left(t\right),x_{i}^{*}\left(t\right)\right)$$

If the importance of each sequence is different, the formula for calculating the GRG is as follows:

(9) $$\varphi \left(x_{r}^{*},x_{i}^{*}\right)=\sum_{t=1}^{m_{2}}w_{t}\gamma \left(x_{r}^{*}\left(t\right),x_{i}^{*}\left(t\right)\right)$$

where $w_{t}$ denotes the weight coefficient of the *t* sequence.

#### 3.2.3 Contribution analysis method

In order to study the influence of process system stability and process parameters on the properties and consistency of tensile samples, contribution analysis method was adopted. The contribution analysis method and process are as follows: [36], [37].

The response value of any performance indicator can be approximated by the parameter variables selected in the multiple regression model. Suppose there are α parameter variables in the experiment ($x_{1},x_{2},\cdots x_{\alpha }$), then the response of any performance indicator can be expressed as:

(10) $$P\left(x_{1},x_{2},\cdots x_{\alpha }\right)=\mu +\sum_{i=1}^{\alpha }Q_{i}\left(x_{i}\right)+\cdots +\sum_{i=2}^{\alpha }\sum_{j=1}^{i-1}R_{\mathrm{ij}}\left(x_{i},x_{j}\right)+\varepsilon$$

where $P\left(x_{1},x_{2},\cdots x_{\alpha }\right)$ represents any performance indicator, $\sum_{i=1}^{\alpha }Q_{i}\left(x_{i}\right)$ is the primary response of the parameter variable, $\sum_{i=2}^{\alpha }\sum_{j=1}^{i-1}R_{\mathrm{ij}}\left(x_{i},x_{j}\right)$ is the cross effect of any two variables, μ represents the constant term and ε represents the error.

The main response function of the regression model can be given by:

(11) $$\sum_{i=1}^{\alpha }Q_{i}\left(x_{i}\right)=\sum_{i=1}^{\alpha }\beta_{i}x_{i}$$

The formula for calculating the contribution value of the variable is:

(12) $$N_{x_{i}}=\frac{100\beta_{i}}{\sum_{i}\left|\beta_{i}\right|}$$

where $N_{x_{i}}$ is the contribution of $x_{i}$, $\beta_{i}$ represents the main response coefficient of $x_{i}$, *i = 1,2,…α.*

### 3.3 Measurements

#### 3.3.1 Tensile testing

The mechanical properties (tensile strength and elongation at break) of micro tensile samples were measured by Meters Industrial System universal testing machine model CMT6103. According to ASTM D638 test standard for plastic tensile samples, tensile samples are measured applying a 500 N load cell with a measurement error of 0.5 % of the indicated value.

#### 3.3.2 Scanning electron microscopy (SEM)

In order to characterize the fracture micro-morphology and defects, scanning electron microscopy (TESCANMIRA LMS) was adopted to observe the fracture surface of tensile samples treated with liquid nitrogen embrittle fracture at low temperature and gold spray.

#### 3.3.3 Gel permeation chromatography (GPC)

The molecular weight was measured by Agilent PL-GPC50 gel permeation chromatograph from the United States. Trichlorobenzene was adopted as the mobile phase at 150℃ and the flow rate was 1 mL/min.

#### 3.3.4 Fourier transformation infrared spectroscopy (FTIR)

Fourier infrared spectroscopy (Thermo Fisher Scientific Nicolet iS20) was adopted to measure and analyze composition of polymer. The spectrum of the sample was scanned between 4000 and 500 cm^−1^.

## 4 Design of experimental

### 4.1 Experimental design of ultrasonic plasticizing UHMWPE raw material preparation

In this study, the ultrasonic amplitude, pressing depth ratio ($T=\frac{a}{b}$, T is pressing depth, a is the ultrasonic sonotrode down press length, b donates the total length of plasticizing cavity), mold temperature and ultrasonic time were screened as the process parameters of ultrasonic tablet. In addition, the ultrasonic amplitude range is 60–90 % (48 μm-72 μm), the pressure range is 6.5/38–8/38, the mold temperature range is 30–90 ℃, and the ultrasonic time is 3–9 s. Among them, the basic process parameters are set as follows: ultrasonic amplitude is 70 % (56 μm), depth of pressure is 7/38, mold temperature is 50 ℃, ultrasonic time is 5 s. All process parameters were taken to four levels and a single factor experiment was carried out, as shown in Table 2.

**Table 2:** The ultrasonic tablet process parameter setting.

Level	Ultrasonic amplitude (μm)	Pressing depth ratio	Mold temperature (℃)	Ultrasonic time (s)
1	48	6.5/38	30	3
2	56	7/38	50	5
3	64	7.5/38	70	7
4	72	8/38	90	9

### 4.2 Screening experiment design of the ultrasonic molded UHMWPE tensile sample

The irregularly shaped particles prepared under the optimal ultrasonic tablet process parameters were applied as the raw materials for ultrasonic plasticizing. Three process parameters, ultrasonic amplitude, plunger speed and mold temperature, were selected as design variables. The UPMIM equipment was applied to conduct the pre-experiment on the tensile sample of UMHWPE, and the molding process parameter window was determined.

### 4.3 Multi-objective optimization experimental design of the ultrasonic molded UHMWPE tensile sample

The reasonable range of ultrasonic amplitude, plunger speed and mold temperature obtained in the pre-experiment was selected as the design variable range of the molding experiment. Orthogonal experiments with 9 experiments were designed with three levels of each process parameter. In order to ensure the reliability of the experimental results, the experiment was repeated 5 times for each group of process parameters in the experiment.

## 5 Results and discussion

### 5.1 The UHMWPE tablet slice analysis and the influence of process parameters on the UHMWPE tablet weight

**Fig. 4:**
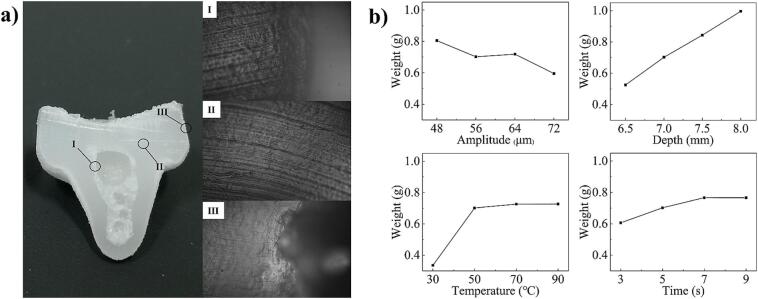
The slice morphology of ultrasonic tablet and the weight of ultrasonic tablet under different process parameters. (a) The morphology for the outer, middle and inner regions of the tablet transverse surface of ultrasonic tablet. (b) The weight and change trend of ultrasonic tablet under different process parameters.

The tablet of ultrasonic molding is shown in Fig. 3b. Moreover, the ultrasonic tablet of the optimal process parameters was sliced, and the structure morphology for the outer, middle and inner regions of the tablet transverse surface was observed, as shown in Fig. 4a. As a result, the UMHWPE powder was completely melted and the interpowder gap disappeared under ultrasonic action. In addition to the huge void present in the center of the ultrasonic tablet, the remaining regions form a solid without defects. This may be due to the rapid melting of the powder under high-frequency ultrasonic vibration, but the gas existing in the particles cannot escape, and eventually a huge trapped gas cavity is formed in the center of the tablet.

In order to further explore the influence of process parameters on ultrasonic compression, the weight of ultrasonic tablet was measured, as shown in Fig. 4b. It is evident that the weight of the ultrasonic tablet varies significantly under diverse process conditions. Specifically, the weight decreased as the ultrasonic amplitude increased. Conversely, the tablet weight saw a significant increase corresponding to higher pressing depth ratios. The weight exhibited an initial increase with rising mold temperature, then plateaued. A marginal increase in weight was observed with prolonged ultrasonic time. The underpinning mechanisms for these observations are as follows: higher amplitudes extend the penetration depth of the ultrasonic energy field, lengthening the tablet. However, this also intensifies the reciprocal friction between the powders, leading to less efficient heat generation and melting, which in turn, results in a narrower diameter at the bottom end of the tablet and consequently, a reduced weight. As the pressing depth ratio of the ultrasonic sonotrode increases, so does the intensity of the ultrasonic energy field, thereby enlarging the tablet weight. Additionally, greater mold temperatures or extended ultrasonic times lead to a larger quantity of the powder melting within the reach of the ultrasonic energy field, thus increasing the tablet's weight. Yet, since neither the mold temperature nor the ultrasonic time can widen the scope of the ultrasonic energy field, the tablet weight ceases to rise upon reaching a certain threshold. Ultimately, the process parameter yielding the greatest weight (an ultrasonic amplitude of 56 µm, a pressing depth of 8/38, a mold temperature of 50 °C, and an ultrasonic time of 5 s) was selected as the optimal scheme for tablet formation. Besides that, the physical property parameters of ultrasonic tablet were further verified by molecular weight determination (Fig. 8g). The results show that it has little effect on molecular weight, and can be used in the subsequent ultrasonic plasticizing experiments.

### 5.2 The process window analysis of screening experiment for ultrasonic molded UHMWPE tensile sample

Table 3 shows the filling state in the Screening experiments of ultrasonic molded UHMWPE tensile sample. In detail, “×” means that at least one of the five experiments could not form a complete tensile sample, and “√” means that a complete tensile sample can be obtained in five experiments. Under the condition that the diameter ratio of the ultrasonic sonotrode to plasticizing cavity is 15 mm: 10 mm, when the ultrasonic amplitude is greater than 60 % (48 μm), the plunger speed is greater than 2 mm/s, and the mold temperature is greater than 60℃, the UHMWPE tensile sample can be fully formed. However, with a diameter ratio of 8 mm: 8 mm, the UHMWPE tensile sample achieves full filling exclusively at an ultrasonic amplitude of 100 % (56.2 μm), with a specific plunger velocity and a mold temperature exceeding 90 °C, as detailed in Table 4. In this Table, the PVP is a combination of plunger rising speed; the UT-UPE represents the raw material of ultrasonic plasticized UMWPE is powder; the C-Cir denotes the raw material of ultrasonic plasticized UMWPE is circular tablet; the C-Irr represents the raw material of ultrasonic plasticized UMWPE is irregular tablet. Therefore, the large diameter ratio of the ultrasonic sonotrode to plasticizing cavity can greatly widen the process window for the complete filling of ultrasonic molded UNHWPE tensile samples.

**Table 3:** The filling state in the pre-experiment of ultrasonic molded UHMWPE tensile sample.

Process parameters	Amplitude (μm)	40 (50 %)				48 (60 %)				56 (70 %)				64 (80 %)			
	Mold temperature (℃)	40	60	80	100	40	60	80	100	40	60	80	100	40	60	80	100
Plunger speed (mm/s)	2	×	×	×	×	×	×	×	×	×	×	×	√	×	×	√	√
	2.5	×	×	×	×	×	√	√	√	×	√	√	√	×	√	√	√
	3	×	×	×	×	×	√	√	√	×	√	√	√	×	√	√	√
	3.5	×	×	×	×	×	√	√	√	×	√	√	√	×	√	√	√

**Table 4:** The filling state in the pre-experiment of ultrasonic molded UHMWPE tensile sample in the comparative literature [26].

Amplitude (μm)	45 (80 %)				50.6 (90 %)				56.2 (100 %)		
PVP (mm/s)	a	b	c		a	b	c		a	b	c
UT-UPE	x	x	x		x	x	x		x	x	x
C-Cir	x	x	x		x	x	√		x	x	√
C-Irr	x	x	x		x	x	√		x	x	√

According to the results of the pre-experiment, the ultrasonic amplitude of 60–80 %, the plunger speed of 2.5–3.5 mm/s, and the mold temperature of 60–100 ℃ were selected as the process parameters of the UHMWPE tensile sample molding experiment. The experimental design is shown in Table 5.

**Table 5:** Experimental design of ultrasonic molded UHMWPE tensile sample.

Level	Amplitude (μm)	Plunger speed (mm/s)	Mold temperature (℃)
1	48	2.5	60
2	56	3	80
3	64	3.5	100

### 5.3 Multi-objective optimization of ultrasonic molded UHMWPE tensile sample

#### 5.3.1 Experimental result

**Fig. 5:**
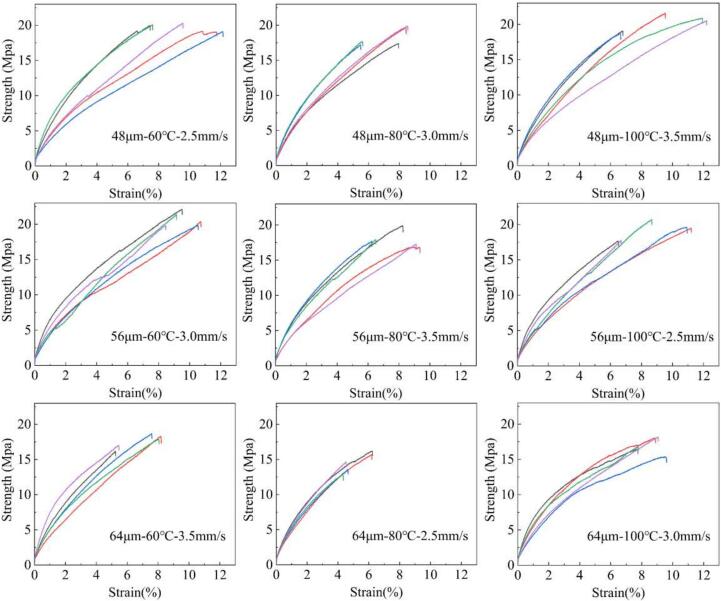
The EB and TS of tensile samples under different process parameters.

**Fig. 6:**
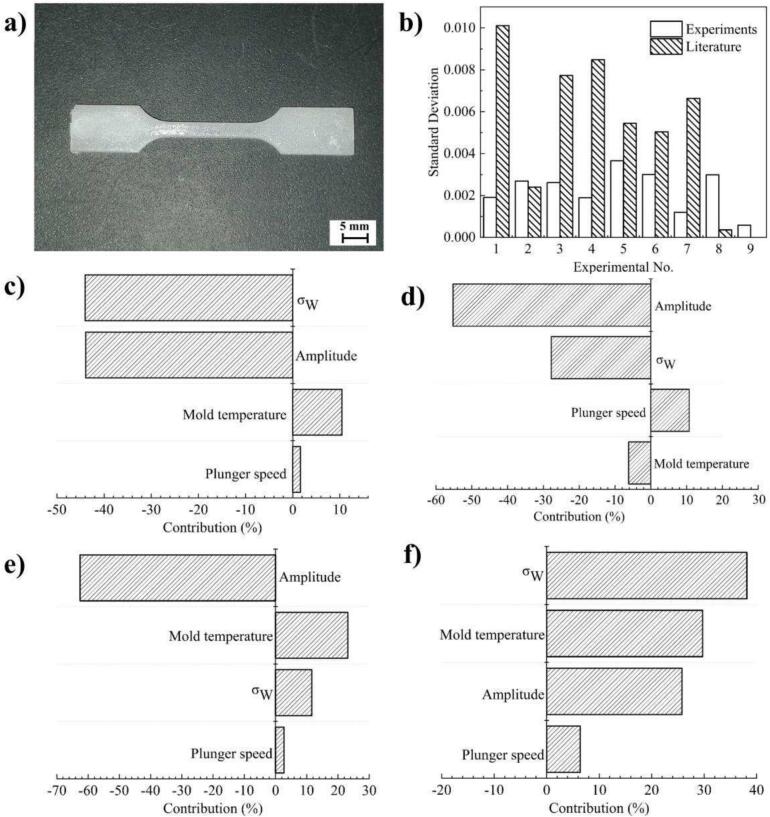
UHMWPE tensile sample, σ_W_ comparison results, mechanical properties and consistency contribution analysis results. a) Ultrasonic molded UHMWPE tensile sample. b) Comparison of the σ_W_ of the tensile sample in this experiment and in Table 5 of the comparative literature [26]. c-f) The contribution of process parameters and ultrasonic system stability to EB, TS, σ_WB_ and σ_TS_.

Fig. 6a shows the UHMWPE tensile sample molded in the experiment. It can be seen that the tensile sample as a whole is milky white, uniform in color, and complete without defects. In addition, the weight of the molded tensile samples was weighed. The elongation at break (EB) and tensile strength (TS) were tested as shown in Fig. 5. Meanwhile, the standard deviation of weight (σ_W_), the standard deviation of elongation at break (σ_EB_) and the standard deviation of tensile strength (σ_TS_) were calculated respectively. The specific process parameters and corresponding property results are shown in Table 6.

**Table 6:** Process parameters and property results of ultrasonic molded UHMWPE tensile samples.

NO.	Process parameters				Property results				
	Amplitude (μm)	Plunger speed (mm/s)	Mold temperature (℃)		EB (%)	TS (MPa)	σ_EB_	σ_TS_	σ_W_
1	48	60	2.5		9.718	19.762	1.975	0.505	0.00191
2	48	80	3		7.202	18.368	1.364	1.173	0.00269
3	48	100	3.5		9.438	20.148	2.391	1.093	0.00262
4	56	60	3		9.68	20.702	0.853	0.885	0.00190
5	56	80	3.5		7.962	17.932	1.369	1.053	0.00365
6	56	100	2.5		8.886	19.048	2.094	1.157	0.00301
7	64	60	3.5		6.912	17.592	1.285	0.914	0.00121
8	64	80	2.5		5.078	14.578	0.882	1.266	0.00298
9	64	100	3		8.796	16.992	0.702	1.035	0.00059

#### 5.3.2 Optimization objectives and grey relational analysis

The product consistency has always been a crucial evaluation indicator for injection molding products. The traditional injection molding process has good stability, and the consistency of the product is usually not considered in the multi-objective optimization. However, the ultrasonic plasticizing microinjection molding process is a strongly coupled process, leading to significant fluctuations in the ultrasonic energy field and resulting in poor product consistency. Therefore, in this study, EB, TS, σ_EB_, σ_TS_ and σ_W_ were all adopted as optimization objectives.

In this study, the mechanical properties of tensile samples are the larger the better, while the fluctuation of mechanical properties of tensile samples is the smaller the better. Therefore, formula (1) is adopted to normalize the original sequence of elongation at break and tensile strength. Formula (2) is used to normalize the original sequence of standard deviation of tensile strength and standard deviation of elongation at break. The normalization results of all sequences are shown in Table 7. Then, formula (4) is adopted to calculate the normalized sequence GRC. In this study, the weights of each optimization objective are the same, so formula (8) is used to calculate GRG. The GRC, GRG and order results of each sequence are shown in Table 8. The results show that the GRG of experiment No.4 is the largest. That is, when the ultrasonic amplitude is 56 μm, the mold temperature is 60 ℃, and the plunger speed is 3 mm/s, the EB, TS, σ_EB_, and σ_TS_ of the tensile samples are the best.

**Table 7:** The normalization results of property for UHMWPE tensile sample.

No.	EB (%)	TS (MPa)	σ_EB_	σ_TS_
Idea	1.000	1.000	1.000	1.000
1	1.000	0.847	0.246	1.000
2	0.458	0.619	0.608	0.122
3	0.940	0.910	0.000	0.227
4	0.992	1.000	0.911	0.500
5	0.622	0.548	0.605	0.280
6	0.821	0.730	0.176	0.143
7	0.395	0.492	0.655	0.462
8	0.000	0.000	0.893	0.000
9	0.801	0.394	1.000	0.304

**Table 8:** The GRC, GRG and order results for UHMWPE tensile sample.

No.	Grey relational coefficient				Grey relational grade	Order
	EB (%)	TS (MPa)	σ_EB_	σ_TS_		
Idea	1.000	1.000	1.000	1.000		
1	1.000	0.765	0.399	1.000	0.633	2
2	0.480	0.567	0.561	0.363	0.394	8
3	0.892	0.847	0.333	0.393	0.493	4
4	0.984	1.000	0.848	0.500	0.666	1
5	0.569	0.525	0.559	0.410	0.413	6
6	0.736	0.649	0.378	0.368	0.426	5
7	0.453	0.496	0.592	0.482	0.404	7
8	0.333	0.333	0.824	0.333	0.365	9
9	0.716	0.452	1.000	0.418	0.517	3

#### 5.3.3 Ultrasonic system stability, mechanical properties and consistency analysis of tensile sample

In order to investigate the stability of ultrasonic system and the influence of process parameters and process stability on the properties and consistency of tensile samples, several evaluation indexes of tensile samples were analyzed. The σ_W_ is applied as the evaluation index to evaluate the stability of the ultrasonic system. The EB and TS are applied as the mechanical properties index. The σ_EB_ and σ_TS_ are adopted as the evaluation index of the consistency for mechanical properties. The influence of the change for ultrasonic sonotrode diameter on the stability for ultrasonic system, the influence of ultrasonic system stability and process parameters on the mechanical properties of tensile samples, and the influence of ultrasonic system stability and process parameters on the consistency of tensile samples were analyzed respectively.

##### 5.3.3.1 The influence of the ultrasonic sonotrode diameter on the stability for ultrasonic system

The tensile sample σ_W_ with the diameter ratio (15 mm:10 mm) of the ultrasonic sonotrode to plasticizing cavity in this study was compared with the diameter ratio (8 mm:8mm) of the ultrasonic sonotrode to plasticizing cavity in literature [26]. The results are shown in Fig. 6b. It can be seen that the σ_W_ of maximum value in this study is 0.00365 and the minimum value is 0.00059, while the σ_W_ of maximum value in literature is 0.01011 and the minimum value is 0.00036. In addition, in terms of numerical value and fluctuation amplitude, the σ_W_ in this study is significantly smaller. Compared with the maximum σ_W_, the literature is 2.8 times that of this study. On the one hand, this is because the increase in the diameter of the ultrasonic sonotrode makes the stiffness larger and the energy field output more stable. On the other hand, with the diameter ratio of the ultrasonic sonotrode to the plasticizing cavity changes bigger, the contact area between the ultrasonic sonotrode and the polymer increases, and the plasticizing power increases. Hence, under the same process parameters, the plasticizing pressure becomes smaller, the melt filling process is more stable, and the molding effect is better.

##### 5.3.3.2 Influence of ultrasonic system stability and process parameters on the mechanical properties of tensile samples

Formula (10)-(12) is adopted to analyze the contribution of ultrasonic system stability and process parameters to the mechanical properties, and the influence rule was shown in Fig. 6c and d. It can be seen that the ultrasonic amplitude and the σ_W_ are negatively correlated to the EB, while the mold temperature and the plunger speed are positively correlated to the EB. The order of contribution from large to small is: σ_W_ (−44.03 %), ultrasonic amplitude (−43.9 %), mold temperature (10.44 %), plunger speed (1.63 %). In contrast, the ultrasonic amplitude, the mold temperature and the σ_W_ are negatively correlated with the TS, while the plunger velocity is positively correlated with the TS. The order of contribution from large to small is: ultrasonic amplitude (−55.26 %), σ_W_ (−27.84 %), plunger speed (10.72 %), mold temperature (−6.18 %). As a result, for the mechanical properties of the tensile sample, the contribution of process parameters accounted for 55.97 %–0.72.16 %, and the contribution of ultrasonic system stability accounted for 27.84 %–44.03 %. With the increase of the ultrasonic amplitude and the σ_W_, the mechanical properties of the tensile sample decreases. The mechanical properties of the tensile sample increases with the increase of the plunger speed. Meanwhile, with the increase of mold temperature, the EB of the tensile sample increases, but the TS decreases. The observed phenomena can be attributed to several factors. As ultrasonic amplitude rises, the impact of the ultrasonic energy field intensifies, leading to more significant polymer molecular chain breakage and oxidation, consequently degrading the properties [24], [25]. With the increase of the σ_W_, the worse the stability of ultrasonic energy field, the worse the performance of the tensile sample. A faster plunger speed means the melt enters the cavity more swiftly, shortening ultrasonic exposure time, and reducing molecular chain damage and oxidation, which enhances performance [24]. With the increase of mold temperature, the crystal type of the tensile sample changes, which leads to the increase of the EB and the decrease of the TS of the sample [26].

##### 5.3.3.3 Influence of ultrasonic system stability and process parameters on the consistency of mechanical properties for tensile sample

The contribution analysis results of the consistency for tensile sample mechanical properties are shown in Fig. 6e and f. It can be seen that the ultrasonic amplitude is negatively correlated with the σ_EB_. And the mold temperature, the σ_W_ and the plunger speed are positively correlated with the σ_EB_. The order of contribution from large to small is: ultrasonic amplitude (−62.56 %), mold temperature (23.12 %), σ_W_ (11.63 %), plunger speed (2.69 %). Moreover, all process parameters have positive correlation to the σ_TS_. The order of contribution from large to small is: σ_W_ (38.12 %), mold temperature (29.68 %), ultrasonic amplitude (25.78 %), plunger speed (6.42 %). The results show that for the consistency of tensile sample mechanical properties, the contribution of process parameters accounts for 61.88 %–88.37 %, and the contribution of ultrasonic system stability accounts for 11.63 %–38.12 %. With the increase of ultrasonic amplitude, the consistency of the EB is better, while the consistency of the TS is worse. Nevertheless, with the increase of the mold temperature and the plunger speed, the consistency of the tensile sample mechanical properties is worse. Meanwhile, the better the stability of ultrasonic system, the better the mechanical properties consistency of sample. The reasons for these phenomena are: with an increase in ultrasonic amplitude, the melt temperature field becomes more uniform, thus increasing EB consistency. However, a higher amplitude also increases the melt temperature, raising the risk of polymer oxidation and worsening the consistency of TS. As mold temperature rises, the molecular chains exhibit more vigorous activity and the crystallization behavior of the melt becomes increasingly intricate, which compromises performance consistency [38]. With the increase of plunger speed, the different size of plasticizing raw material will lead to a high dynamic change of plasticizing pressure, which will increase the fluctuation of ultrasonic energy field. As a result, the performance consistency decreases [39], [40]. Similarly, with the decrease of the stability of the ultrasonic system, the greater the fluctuation of the ultrasonic energy field, and the consistency of the performance decreases.

In summary, whether it is tensile sample mechanical properties or consistency, the influence of process parameters is dominant, and the stability of ultrasonic system is also a factor that cannot be ignored. Therefore, when ultrasonic molded products, under the condition of improving the stability of the ultrasonic system, the needs of product mechanical properties and consistency should be considered comprehensively, and a balance should be found in the process parameters, so that the mechanical properties and consistency are in an acceptable range.

### 5.4 Characterization and comparative analysis of UHMWPE tensile sample

**Fig. 7:**
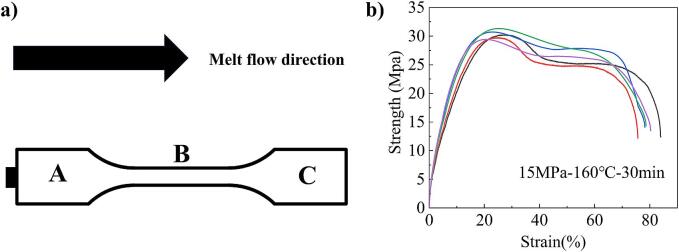
The region division of tensile sample and the mechanical properties of compression molded tensile samples. (a) The ABC division of three regions for UHMWPE tensile sample. (b) The mechanical properties measurement results of compression molded tensile samples.

At present, compression molding is one of the main forming processes of UHMWPE and is widely used [41], [42]. In order to further study the feasibility of ultrasonic molding in UHMWPE forming, the mechanical properties, micro-morphology, molecular weight and composition of UHMWPE tensile samples were measured, and compared with compression molded UHMWPE tensile samples. Three regions of ABC are defined on the tensile sample (Fig. 7a). Region A is the region near the gate in the tensile sample, region B is the middle region of the tensile sample, and region C is the region at the far end of the gate in the tensile sample. Moreover, the ultrasonic molded UHMWPE tensile samples were obtained by using the best process parameters. The compression molded UHMWPE tensile samples were obtained by using the process parameters recommended from the supplier (pressure 15 MPa, mold temperature 160℃, time 30 min).

In the mechanical properties representation, 5 groups of ultrasonic molded and compression molded tensile samples were measured respectively, and the average values of EB and TS were taken as measurement results. In the characterization of the micro-morphology, the B region of the tensile sample is more representative, so the B region of the ultrasonic molded and compression molded tensile samples was scanned. According to previous studies [26], the molecular weight in the C region of the tensile sample changed the most during ultrasonic molding. The material properties in each region of the compression molded tensile sample was not different. Therefore, the molecular weight of the ultrasonic plasticized material, the C region of the tensile sample for ultrasonic molding and the arbitrary region of the tensile sample for compression molding were measured respectively. Each region of the ultrasonic molded tensile sample experiences different ultrasonic intensity, which leads to differences in composition. Therefore, the ABC three regions of the ultrasonic molded tensile sample and any region of the compression molded tensile sample were measured during the characterization of the composition. The characterization schemes of tensile samples and corresponding measurement regions are shown in Table 9. “-” means not measured. “All” represents the entire tensile sample. “Any” denotes any region.

**Table 9:** The characterization schemes of tensile samples and corresponding measurement regions.

Molding method	Type	Measuring region			
		Tensile testing	SEM	GPC	FTIR
Ultrasonic	Ultrasonic plasticizing raw material	−	−	Any	−
	tensile sample	All	B (fracture surface)	C	A, B and C
Compression	tensile sample	All	B (fracture surface)	Any	Any

#### 5.4.1 Mechanical properties

Fig. 7b shows the measurement results of mechanical properties for compression molded tensile samples. Table 10 shows the comparison of mechanical properties for tensile sample under different processes. It can be seen that the EB of the tensile sample obtained by compression molding is 79.36 % and the TS is 30.39 MPa. When the diameter ratio of the ultrasonic sonotrode to plasticizing cavity is 15 mm: 10 mm, the EB of the tensile sample obtained by ultrasonic molding is 9.68 % (about 12.2 % of the compression molding), and the TS is 20.7 MPa (about 68.11 % of the compression molding). However, the existing research results show that when the diameter ratio of the ultrasonic sonotrode to plasticizing cavity is 8 mm: 8 mm, the EB of the ultrasonic molded tensile sample is reduced to 5.56 % of the compression molding process, and the TS is increased to 136.54 % of the compression molding [26]. The results show that the large diameter ratio of the ultrasonic sonotrode to plasticizing cavity increases the EB of ultrasonic molded tensile samples from 5.56 % to 12.2 %, while the TS decreases from 136.54 % to 68.11 %. This is because the large diameter ratio increases the polymer contact area of the ultrasonic sonotrode, increasing filling capacity and reducing plasticizing pressure. With the increase of filling capacity, the exposure time of melt to ultrasonic decreases, the break of molecular chain decreases, and the EB increases. However, the reduction of plasticizing pressure increases the ultrasonic cavitation effect, resulting in a decrease in TS [24], [43]. In order to further explore the causes of this phenomenon, the SEM, the GPC and the FTIR were applied.

**Table 10:** Measurement results of mechanical properties of UHMWPE tensile samples by ultrasonic molding and compression molding.

Comparison	Technology	EB (average) (%)	TS (average) (MPa)
This study	Compression molding	79.36	30.39
	Ultrasonic molding	9.68	20.70
	Ratio	12.20	68.11
Literature [26]	Compression molding	3.60	19.10
	Ultrasonic molding	0.20	26.08
	Ratio	5.56	136.54

#### 5.4.2 SEM

**Fig. 8:**
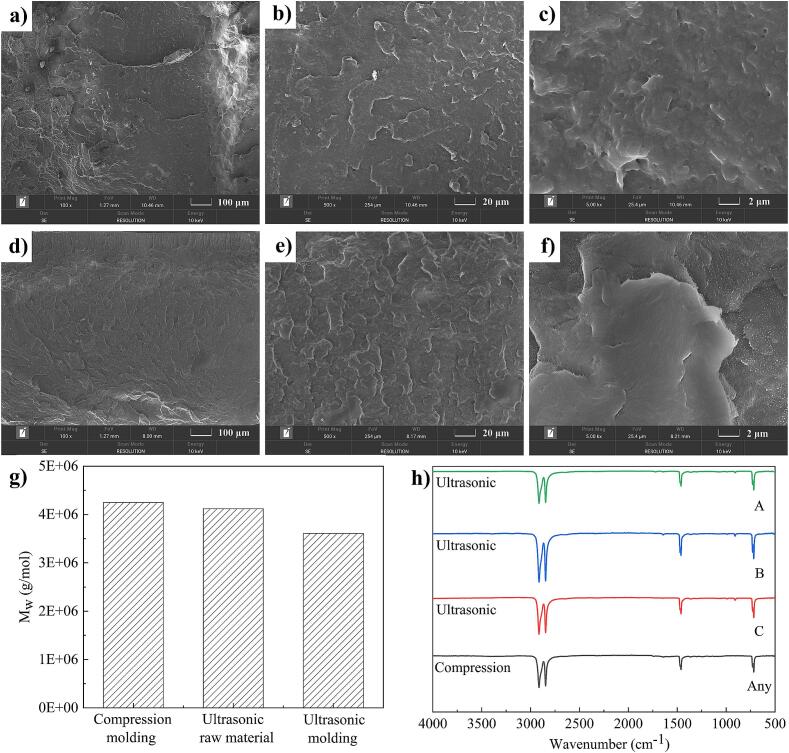
The micro-morphology, molecular weight and FTIR spectra of UHMWPE tensile sample. (a), (b), (c) and (d), (e), (f) The fracture surface micro-morphology of the B region on the tensile sample for compression molding and ultrasonic molding after 100 times, 500 times and 5000 times magnification. (g) The molecular weight measurement results of compression molded tensile sample, ultrasonic plasticizing raw material and C region for ultrasonic molded tensile sample. (h) The FTIR spectra of ABC three regions of ultrasonic molded tensile sample and any region of compression molded tensile sample.

Fig. 8a, b, c and d, e, f show the fracture surface micro-morphology of the B region on the tensile sample for compression molding and ultrasonic molding after 100 times, 500 times and 5000 times magnification, respectively. On the whole, the fracture surface of the tensile sample prepared by compression molding or ultrasonic molding have no obvious porosity, cracks and other defects. Under the magnification of 5000, the fracture surface of the compression molded tensile samples was uneven and a few micro-pores appeared (Fig. 8c). Although the UHMWPE powder melts under high pressure and high temperature, the fuzzy lamination boundary of the powder can still be observed. However, the fracture surface of the ultrasonic formed tensile samples was flat and smooth. The UHMWPE powder was completely melted, and the powder boundary completely disappeared (Fig. 8f). Therefore, compared with compression molding technology, the UPMIM technology can melt UHMWPE powder better and show better microscopic morphology.

#### 5.4.3 GPC

The molecular weight results of UHMWPE under different processes are shown in Fig. 8g. It can be seen that the molecular weight in order from large to small is compression molded tensile sample, ultrasonic plasticized raw material and ultrasonic molded tensile sample, which are 4.25*10^6^, 4.12*10^6^ and 3.61*10^6^ respectively. The results show that the molecular weight of the ultrasonic plasticizing raw material decreased by 0.31 % compared with that of the compression molded tensile sample. The molecular weight of the ultrasonic molded tensile sample decreased by 15.07 % compared with that of the compression molded tensile sample. This is because when UHMWPE ultrasonic plasticizing raw materials are prepared, the plasticizing pressure is small, the friction heat between solid powders is dominant, and the inter-chain thermal movement is low. As a result, the molecular weight drops less. During the ultrasonic molding of UHMWPE tensile samples, the melt of UHMWPE tensile sample was subjected to greater plasticizing pressure, and the ultrasonic effect was enhanced. After the powder melting, the molecular chain unfolds, and the strong ultrasonic action causes the bond between the molecules to break. Therefore, the molecular weight drops more. However, compared with the results of at least about 61.90 % reduction in molecular weight of ultrasonic molded tensile samples in literature [26], the large diameter ratio of the ultrasonic sonotrode to plasticizing cavity adopted in this study can greatly reduce the reduction in molecular weight.

#### 5.4.4 FTIR

Fig. 8h shows the FTIR spectra in three regions of compression molded and ultrasonic molded tensile samples. It can be seen that except for the difference in the characteristic peaks at 908 cm^−1^, the other positions are the same. Specifically, no characteristic peak appeared at 908 cm^−1^ for tensile samples of compression molding, while the characteristic peak existed at 908 cm^−1^ for tensile samples of ultrasonic molding. The signal intensity in descending order is C region, B region and A region. As well known that the characteristic peak at 908 cm^−1^ indicates the presence of vinyl, proving that there is a break in the chain of UHMWPE during the molding process. Besides that, the spectrogram of other area that the tensile samples of ultrasonic molding and compression molding do not show the characteristic peak of oxidative degradation. The results show that the ultrasonic action could lead to the break of the molecular chain of UHMWPE, which corresponded to the reduction of molecular weight, and there was no obvious oxidative degradation phenomenon in this study. However, the study in literature [26] showed that ultrasonic action could not only lead to oxidative degradation of UHMWPE, but also break the molecular chain. Therefore, the large diameter ratio of the ultrasonic sonotrode to plasticizing cavity can inhibit the oxidative degradation of UHMWPE and reduce the breakage of molecular chain. This is because the large diameter ratio increases the polymer contact area of the ultrasonic sonotrode, increases the filling capacity, and reduces the residence time of the melt under ultrasonic action. Moreover, the greater signal intensity in region C is due to the fact that the melt in this region is exposed to ultrasound for a relatively longer time and the intensity of ultrasonic action is greater, which makes the molecular chain break more serious. Specifically, once the melt streams into the cavity during ultrasonic processing, it is no longer directly affected by ultrasound. Thus, the molecular chain breakage is greater in the region exposed to ultrasonication for the longer period and greater intensity before filling the cavity. In the UPMIM process, the earliest plasticized melt is filled into region C. Due to the raw material's low initial temperature, the plasticization and melting require more time, resulting in more extended ultrasonication in region C. Meanwhile, the plasticizing pressure at this stage increased sharply, and the ultrasonic action intensity also increased sharply [17]. After the raw materials in region A and region B are preheated for plasticizing melting in the previous region, the difficulty of plasticizing melting is reduced, the plasticizing pressure is also reduced, and the corresponding ultrasonic acting time and intensity are also greatly reduced. Consequently, the chain scission in regions A and B is less pronounced than that in region C.

## 6 Conclusion

In this study, the feasibility of ultrasonic molding for UHMWPE was thoroughly investigated. The study involved the utilization of ultrasonic technology for tablet preparation, implementation of the UPMIM technique featuring a large diameter ratio of the ultrasonic sonotrode to the plasticizing cavity, the usage of orthogonal experimental design and GRA for multi-objective optimization, employment of contribution analysis to quantitatively assess factors affecting product consistency, and application of tensile testing, SEM, GPC, and FTIR for performance validation. The conclusions obtained are as follows:

1. Ultrasonic technology can effectively convert UHMWPE powder into suitable plasticizing raw material for UPMIM, with minimal change in properties (molecular weight reduction by 0.31 %).

2. The optimal mechanical properties and consistency of UHMWPE tensile samples in this study are achieved with an ultrasonic amplitude of 56 μm, a mold temperature of 60 °C, and a plunger speed of 3 mm/s.

3. A greater diameter ratio of the ultrasonic sonotrode to the plasticizing cavity significantly enhances the process window for the complete filling of UHMWPE tensile samples, increasing the ultrasonic system's filling stability by approximately 1.8 times.

4. The contribution of process parameters to the mechanical properties and consistency of UHMWPE tensile samples ranges from 55.97 % to 88.37 %, while the stability of the ultrasonic system contributes between 11.63 % and 44.03 %, indicating that the effect of ultrasonic system stability is substantial and should not be overlooked.

5. There is an observed decline in the performance of ultrasonically molded UHMWPE tensile samples. Nonetheless, using a larger diameter ratio for the ultrasonic sonotrode relative to the plasticizing cavity can mitigate oxidative degradation and minimize molecular chain breakage. Comparative results show that this approach improves the EB of ultrasonically molded tensile samples from 5.56 % to 12.2 % and decreases the TS from 136.54 % to 68.11 %.

## CRediT authorship contribution statement

**Zhiying Shan:** Writing – review & editing, Writing – original draft, Methodology, Formal analysis, Data curation. **Xingbo Qin:** Writing – review & editing, Methodology, Formal analysis, Data curation. **Hang Li:** Writing – original draft, Supervision, Resources, Project administration, Investigation. **Yanghui Xiang:** Supervision, Resources, Project administration, Investigation. **Wangqing Wu:** Writing – review & editing, Supervision, Resources, Project administration, Methodology, Investigation, Funding acquisition, Conceptualization.

## Declaration of competing interest

The authors declare that they have no known competing financial interests or personal relationships that could have appeared to influence the work reported in this paper.
